# Space-Air-Ground Integrated Mobile Crowdsensing for Partially Observable Data Collection by Multi-Scale Convolutional Graph Reinforcement Learning

**DOI:** 10.3390/e24050638

**Published:** 2022-05-01

**Authors:** Yixiang Ren, Zhenhui Ye, Guanghua Song, Xiaohong Jiang

**Affiliations:** 1 School of Aeronautics and Astronautics, Zhejiang University, Hangzhou 310027, China; yixiangren@zju.edu.cn; 2 College of Computer Science and Technology, Zhejiang University, Hangzhou 310027, China; zhenhuiye@zju.edu.cn (Z.Y.); jiangxh@zju.edu.cn (X.J.)

**Keywords:** mobile crowdsensing, deep reinforcement learning, UAV control, graph network, maximum-entropy learning

## Abstract

Mobile crowdsensing (MCS) is attracting considerable attention in the past few years as a new paradigm for large-scale information sensing. Unmanned aerial vehicles (UAVs) have played a significant role in MCS tasks and served as crucial nodes in the newly-proposed space-air-ground integrated network (SAGIN). In this paper, we incorporate SAGIN into MCS task and present a *Space-Air-Ground integrated Mobile CrowdSensing* (SAG-MCS) problem. Based on multi-source observations from embedded sensors and satellites, an aerial UAV swarm is required to carry out energy-efficient data collection and recharging tasks. Up to date, few studies have explored such multi-task MCS problem with the cooperation of UAV swarm and satellites. To address this multi-agent problem, we propose a novel deep reinforcement learning (DRL) based method called *Multi-Scale Soft Deep Recurrent Graph Network* (ms-SDRGN). Our ms-SDRGN approach incorporates a multi-scale convolutional encoder to process multi-source raw observations for better feature exploitation. We also use a graph attention mechanism to model inter-UAV communications and aggregate extra neighboring information, and utilize a gated recurrent unit for long-term performance. In addition, a stochastic policy can be learned through a maximum-entropy method with an adjustable temperature parameter. Specifically, we design a heuristic reward function to encourage the agents to achieve global cooperation under partial observability. We train the model to convergence and conduct a series of case studies. Evaluation results show statistical significance and that ms-SDRGN outperforms three state-of-the-art DRL baselines in SAG-MCS. Compared with the best-performing baseline, ms-SDRGN improves 29.0% reward and 3.8% CFE score. We also investigate the scalability and robustness of ms-SDRGN towards DRL environments with diverse observation scales or demanding communication conditions.

## 1. Introduction

In the past few years, Mobile Crowdsensing (MCS [1,2]) has rapidly become a popular research paradigm for large-scale information gathering and data sensing, which is an essential solution for the construction of smart cities or the Internet of Things [3]. In general, an MCS task consists of several stages: mobile sensing, crowd data collection, and crowdsourced data processing [4]. The traditional human-centric MCS paradigm relies on the perception capabilities of a large crowd of citizens’ mobile devices, such as mobile phones, wearable devices or portable sensors. Compared with ordinary sensing networks, a human-centric MCS system makes full use of human intelligence for large-scale sensing purposes. However, the major challenge to traditional MCS lies that, users may be reluctant to participate in the MCS system for privacy and security concerns.

With the help of high-precision embedded sensors and path planning algorithms [5], smart unmanned vehicles, including automated guided vehicles (AGVs) and unmanned aerial vehicles (UAVs), are gradually taking the place of human participants for data collection. A swarm of intelligent unmanned vehicles can perform collaborative sensing tasks round-the-clock [6,7], or even cooperate with humans [8]. Among all kinds of unmanned vehicles, UAVs have better maneuverability and versatility compared to ground vehicles. Hence, UAV-based MCS technology can achieve large-scale, high-quality, long-term, and in-depth data collection in diverse real-world scenarios, such as efficient area coverage [9,10], smart city traffic monitoring [11,12], field search and rescue [13], post-disaster relief [14], communication support [15,16], reconnaissance in future wars [17], etc.

As the rapid developments and applications of modern network technologies [18,19], several studies have dug deep into heterogeneous networking and proposed an architecture called Space-Air-Ground Integrated Network (SAGIN [20,21]). SAGIN interconnects space, air, and ground network segments using different networking protocols. Satellite-based networks in space could provide global yet fuzzy observations of large-scale areas, but have some propagation delay due to the operating orbits and long communication ranges. Aerial networks, such as Flying Ad-Hoc Network (FANET [22]), have high mobility and self-organizing ability, but their performance are commonly constrained by unstable connections or dynamic network topology [23]. Ground networks have low transmission latency and efficient power supply, while they cannot maintain network coverage in certain remote areas.

**Figure 1:**
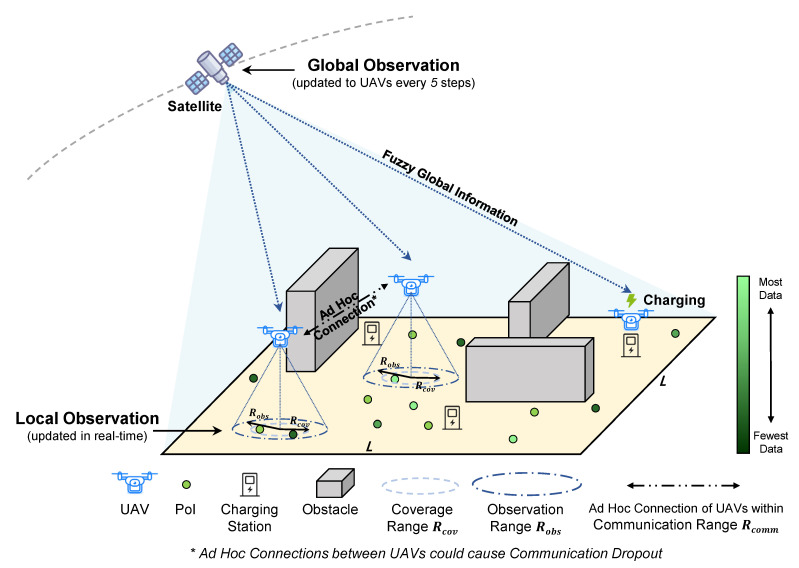
Proposed SAG-MCS Scenario Schematic.

In this paper, we employ the concept of SAGIN into the data collection task, and present a new MCS framework with a collection of UAVs, ground nodes and satellites, namely *Space-Air-Ground integrated Mobile CrowdSensing (SAG-MCS)*. In SAG-MCS scenario, a UAV swarm is used to cooperate autonomously and fly above an area with multiple Points of Interest (PoIs) for coverage and sensing. As illustrated in Figure 1, UAV agents can partially observe ground information using embedded sensors within a fixed observation range. They also have access to fuzzy global information periodically from remote sensing satellites in space, which contains ambiguous locations of PoIs and other agents. As the coverage range is set smaller than the observation radius, UAVs should get close enough to the observed PoIs for valid data collection. Based on the FANET, UAV pairs that within maximum communication range can interconnect together and share current states and observations using Wi-Fi, Bluetooth or LoRa. We consider communication dropout would occur inevitably during such aerial ad-hoc network connections. As for energy consumption, due to the limitations of the rotor power efficiency and the onboard battery capacity, we set all UAVs with limited battery attributes as energy constraints. Several charging stations and barriers are deployed in the SAG-MCS simulation scenario as well. The UAV swarm is required to avoid collision with obstacles when performing data collection and flight path planning tasks, and makes proper decisions to go for charging before their batteries run out. On arrival at the charging stations, UAVs can transfer the data collected and batteries will be replaced.

On the whole, this paper endeavours to propose a decision-making model for UAVs, which are powered by limited onboard batteries and distributed charging stations, to energy-efficiently and persistently sense and collect PoIs on the ground. The multi-UAV swarm shall perform actions according to local airborne observations and global observations from satellites. The overall optimization objective of the UAV swarm is to maximize the data coverage and geographical fairness among all PoIs, and minimize the power consumed during flying or battery charging.

For such an MCS task that has multiple complex objectives, existing approaches that modeling MCS as an optimization problem is no longer effective. However, recently well-explored Deep Reinforcement Learning (DRL) could be a feasible solution. It has achieved great performances in several game-playing tasks [24] or path planning problems [25]. Based on powerful deep neural networks, DRL models can extract more complicated features of higher dimensions from environmental states, thereby can optimize action policies to achieve different objectives. For multi-agent systems such as our SAG-MCS, typical methods that take the whole system as a single agent cannot guarantee promising results, while recent studies on Multi-Agent Deep Reinforcement Learning (MADRL) focus on controlling multiple agents in a fully distributed manner. The action strategy of each agent in MADRL depends on not only the interaction with the environment, but also other agents’ actions, observations, etc.

### Contributions

To this end, this paper formulates the problem as a Partially Observable Markov Decision Process (POMDP) and proposes a stochastic MADRL algorithm in SAG-MCS environment, to perform data collection and task allocation simultaneously. The main contributions of this article are summarized as follows:

1. We design a realistic SAG-MCS environment with obstacles and charging stations for simulation. To further enhance exploration of the global area, the UAV swarm can acquire multi-source observation inputs from embedded sensors and satellites.
2. We propose a DRL algorithm based on graph attention mechanism, namely *Multi-Scale Soft Deep Recurrent Graph Network (ms-SDRGN)*. It integrates a multi-scale convolutional encoder to process different sizes of observations. This method also utilizes graph attention network (GAT [26]), gated recurrent unit (GRU [27]), as well as a maximum entropy method.
3. Although UAV swarm can receive parts of global observations from satellites, SAG-MCS is formulated as a practical partially-observed problem, and UAVs cannot have access to the overall system states during training. Therefore, we propose a heuristic reward function that only utilizes local observed information, but manages to train the UAV swarm to properly act for optimizing several global metrics.
4. We have designed and conducted several simulation case studies to verify the effectiveness of our stochastic MADRL method and reward function. Additionally, we validate the robustness of the trained model and the multi-scale CNN encoder under different communication conditions and at different environment scales.

The remaining part of this paper proceeds as follows: Section 2 reviews the related research efforts about MCS and DRL approaches. Section 3 introduces the SAG-MCS problem definition and the 2D simulation environment in detail. Section 4 presents the proposed solution ms-SDRGN for SAG-MCS problem. We introduce simulation settings and present the experimental results and analysis in Section 5. Then, Section 6 discusses the practical implementation issues and limitation of the proposed approach. Finally, conclusions are made in Section 7.

## 2. Related Works

In this section, we review the literature related to mobile crowdsensing problem, DRL approaches for multi-agent systems, and the joint studies of these two topics.

Threat to validity [28,29]: For this review, we have used multiple strings to search and identify relevant literature in recent decade, such as ‘UAV swarm and mobile crowdsensing’, ‘multi-task allocation and mobile crowdsensing’ and ‘multi-agent deep reinforcement learning’. Google Scholar is used for forward searches and most of the related works are retrieved from five databases: IEEE Xplore, SpringerLink, Web of Science, ScienceDirect and Arxiv.

### 2.1. Multi-Task Allocation for Mobile Crowdsensing

MCS scenarios usually have multiple constraints and objectives. One of the key issues is how to perform task allocation, or how to choose appropriate action strategies for different tasks. The main tasks of SAG-MCS are data collection by covering PoIs and energy management by keeping batteries charged. UAVs need to automatically select action strategies to meet the data collection requirements under the energy-efficiency constraint. Solving such multi-agent task allocation is an NP-hard problem, and the related research is still in a relatively early stage. Feng et al. [30] utilized dynamic programming for path planning in UAV-aided MCS and used Gale-Shapley-based matching algorithm to allocate different tasks for agents. Wang et al. [31] modeled multi-task allocation as a dynamic matching problem, then proposed a multiple-waitlist based task assignment (MWTA) algorithm. In addition, several surveys of task allocation have demonstrated the effectiveness of heuristic algorithms. Hayat et al. [13] proposed a genetic algorithm approach to get the minimum task completion time for UAV path planning. Similarly, Xu et al. [32] formulated this problem as a specific mathematical model, and tried to minimize incentive cost under the constraint of sensing quality based on greedy algorithms and genetic algorithms.

### 2.2. Deep Reinforcement Learning (DRL) for Multi-Agent Systems

In multi-agent systems, Reinforcement Learning (RL) generally targets at problems of agents sequentially interacting with local environment. At timestep *t*, the environment is at state $s_{t}$ and agent *i* obtains a observation $o_{t}^{i}$. Then, agent *i* selects and executes an action $a_{t}^{i}$ based on $o_{t}^{i}$, and then gets a reward $r_{t}^{i}$ from the environment. In POMDP, agents cannot directly perceive the underlying states and $o_{t}^{i}$ is not equal to $s_{t}$. The objective of RL is to learn a policy $\pi_{i}\left(a_{i}|o_{i}\right)$ for agent *i*. The policy is expected to maximize the discounted reward $E\left[R_{t}\right]=E\left[\sum_{k=0}^{\infty }\gamma^{k}r_{k}^{i}\right]$, with a discounted factor γ∈[0,1].

Currently, DRL methods have achieved state-of-the-art performance in various RL tasks [24,25], and can be categorized into value-based or policy-based ones. In this paper, we adopted the value-based method. Deep Q-learning (DQN [24]) is one of the most vital value-based DRL approaches. Based on Q-learning, DQN uses deep neural networks to learn a Q-value function Q(o,a), which could estimate the expected reward return $E[R_{t}]$ and be recursively updated. DQN regards the action with biggest Q-value as the most optimal policy $\pi^{{}^{\prime }}\left(s\right)=\arg\max Q^{\pi }\left(o,a\right)$, and selects it to interact with the environment. In addition, DQN integrates fixed target network and experience replay methods to make the training process more efficient and stable [33]. Specifically, the Q-value function Q(o,a) is updated through minimizing the Q-loss function as:

(1) $$Q_{loss}={\left(r_{t}+\max_{a_{t+1}}Q^{\prime }\left(o_{t+1},a_{t+1}\right)-Q\left(o_{t},a_{t}\right)\right)}^{2}.$$

where *Q* is the learned network and $Q^{\prime }$ is the target network. Note that the policies learned by DQN are deterministic, therefore DQN should be trained with action policies such as ϵ−greedy to enhance exploration.

Compared with classical heuristic algorithms, agents can learn a strategy more efficiently and independently through DRL algorithms, so as to achieve multiple objectives in the sensing area simultaneously.

### 2.3. DRL Methods for UAV Mobile Crowdsensing

To date, several studies have investigated the application of DRL algorithms in the UAV Mobile Base Station (MBS) scenario, which is a sub-topic of MCS. In the UAV MBS scenario, a swarm of UAV serve as mobile base stations to provide long-term communication services for ground users. Liu et al. [15] proposed a DRL model based on Deep Deterministic Policy Gradient (DDPG [34]) to provide the long-term communications coverage in the MBS scenario. Further, Liu et al. [16] implemented DDPG in a fully distributed manner.

Different from policy gradient methods, Dai et al. [35] applied Graph Convolutional Reinforcement Learning (DGN [36]) in MBS. They modeled the UAV swarm as a graph, and used Graph Attention Network (GAT [26]) as a convolution kernel to extract adjacent information between neighboring UAVs. To further explore the potential of graph networks, Ye et al. [37] designed a FANET based on GAT, named GAT-FANET, allowing two adjacent UAV agents within the communication range to communicate and exchange information at low costs. This work also applied Gated Recurrent Unit (GRU) as a memory unit to record and process long-term temporal information from the graph network.

On the basis of MBS, Liu et al. [38,39] took practical factors such as obstacles and charging stations into consideration in the UAV MCS scenario. Based on the actor-critic network of DDPG, their DRL models used CNN to extract observed spatial information, and deployed a distributed experience replay buffer to store previous training information. Piao et al. [40], Dai et al. [41] and Liu et al. [38] utilized the concept of the Long Short-term Memory (LSTM [42]) network to store sequential temporal information of previous interaction episodes. As a specific application of MCS, Dai et al. [41] designed an approach for mobile crowdsensing, where mobile agents are required to retrieve data and refresh the sensors distributed in the city, with limited storage capacities of the sensors. Wang et al. [43] proposed a more practical and challenging 3D MCS scene for disaster response simulation, where the UAVs’ action space had been expanded to three dimensions.

Compared with the UAV MBS and MCS works mentioned above, this paper proposes a more complicated and promising SAG-MCS scenario, which incorporates global and local observations from space and air, respectively, and encourages UAVs to interact with charging stations as ground nodes. While [38,39,40,41] proposed multi-UAV MCS scenarios and used policy-based DRL methods as solutions which utilized LSTM to store temporal information of MCS systems, our approach selects the value-based method based on DQN and uses GRU as the memory unit, which performs similarly to LSTM but is more computationally efficient [44]. Furthermore, when most MADRL studies about MCS solved the problem with deterministic policies, our method learns a stochastic policy following Ye et al. [37] to improve robustness.

## 3. System Model and Problem Statement

In this section, we design a partially observable space-air-ground integrated MCS system, with space-based remote sensing satellites and an aerial UAV swarm jointly performing the MCS task. We define the problem and present the 2D simulation system model specifically. Then, we describe the design of evaluation metrics.

### 3.1. System Model

As illustrated in Figure 1, the SAG-MCS scenario is simplified to a 2-dimensional continuous square area with the size of L×L pixels. The simulation area has fixed borders and multiple obstacles that UAVs cannot fly over. We assume that there are a set K≜{k∣k=1,2,…,K} of PoIs, and each PoI is assigned a certain data amount d(k),∀k. Note that PoIs are regarded as persistent information nodes and are not going to disappear after coverage. Additionally, we consider a set C≜{c∣c=1,2,…,C} of charging stations and a set B≜{b∣b=1,2,…,B} of round and rectangular obstacles. At the beginning of each simulation episode, the locations of all the PoIs, charging stations, and obstacles are randomly distributed in the 2D map. Each PoI’s data amount d(k),∀k is randomly assigned in a certain range as well, but the total data volume $\Sigma_{k}d\left(k\right)$ of different episodes remains consistent.

Let U≜{u∣u=1,2,…,U} be *U* UAV agents deployed in the simulation area, where the UAVs can perform continuous and horizontal flying movements at a fixed altitude. We define $R_{obs}$ as the observation range, and $R_{cov}$ as the coverage range or sensing range of each UAV. Arbitrary UAV can observe the local map within the radius $R_{obs}$ in real-time and receive $L_{sat}\times L_{sat}$ fuzzy global map captured by satellites every some timesteps. Any PoI *k* within a UAV’s $R_{cov}$ is recognized as covered and all its data d(k) is collected once at each timestep *t*. Note that $R_{cov}$ is smaller than $R_{obs}$, as UAVs can only collect data when approaching to PoIs, but they can observe a wider range of area in general. Moreover, we consider the UAV swarm can autonomously form the ad-hoc network, and each pair of agents can be interconnected within communication range $R_{comm}$ and exchange observed information for joint decision making. Considering the delays and packet losses in real-world ad-hoc networking, we set a communication dropout probability *p* between adjacent UAV nodes in training and evaluation. As for the energy consumption, we set the onboard battery status ϕ(u)∈[0,100%],∀u.

For each simulation episode, the data collection task in SAG-MCS scenario will last for *T* timesteps in total. Each UAV’s position is randomly assigned and their batteries are fully-charged in the beginning. At each timestep *t*, UAV *u* can obtain local observation from embedded sensors; while every few timesteps, it can obtain fuzzy global observation from the satellite. Using the multi-scale observations ${\left\{o_{t}^{u}\right\}}_{u\in U}$, UAV *u* performs an action ${\left\{a_{t}^{u}\right\}}_{u\in U}$. We set the battery ϕ(u) consumed at timestep *t* as ${\left\{e_{t}^{u}\right\}}_{u\in U}$, which is determined by the current flying speed ${\left\{v_{t}^{u}\right\}}_{u\in U}$ and will be introduced in Section 3.4. When flying close to charging stations, their batteries will be fully charged in next timestep, simulating the real-world battery replacement process on the ground.

### 3.2. Observation Space

**Figure 2:**
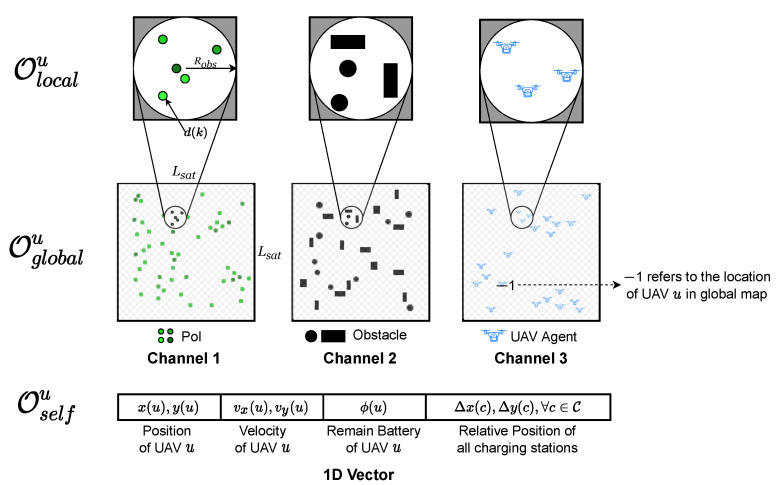
The observation space of UAV *u* in SAG-MCS.

In SAG-MCS, each UAV agent *u* can obtain the multi-scale observation ${\left\{o_{t}^{u}\right\}}_{u\in U}$ at timestep *t* from different sources, as introduced in Section 3.1. In Figure 2, we formulate the observation space with three elements: $O≜{\left\{o_{t}^{u}=\left(O_{local}^{u}\;,O_{global}^{u}\;,O_{self}^{u}\right)\right\}}_{\forall u\in U}$.

(1) Local observation $O_{local}$ from embedded sensors: UAV can observe local information within a circle of radius $R_{obs}$ in real-time, centering on itself. Let $O_{local}≜{\left\{o_{l}^{u}=\left(O_{local}^{u,1},O_{local}^{u,2},O_{local}^{u,3}\right)\right\}}_{\forall u\in U}$ denotes local observation space, which consists of three 2D vector channels. The first channel contains the data amounts and distribution of surrounding PoIs. We set the data value d(k) as the corresponding pixel value if it refers to PoI *k*, otherwise 0. The second channel contains the locations of obstacles relative to the UAV, where we set pixel value 1 for coordinates of obstacles, otherwise 0. The third channel includes the locations of other UAVs within $R_{obs}$. In addition, we define pixel value 1 for coordinates of UAV agents as well, otherwise 0.

(2) Global observation $O_{global}^{u}$ from satellites: Every *n* timesteps, satellites will capture fuzzy global observation and transmit the information to all UAVs. As shown in Figure 2, $O_{global}^{u}$ consists of three 2D channels with reduced size of $L_{sat}\times L_{sat}\left(L_{sat}<L\right)$, which cannot provide precise locations of the environment elements globally. We define $O_{global}≜{\left\{o_{g}^{u}=\left(O_{global}^{u,1},O_{global}^{u,2},O_{global}^{u,3}\right)\right\}}_{\forall u\in U}$ in absolute positioning coordinates. The encoding method for global observation is nearly the same as local observation, except in the third channel of UAV locations, we set −1 as the corresponding pixel value if it refers to the absolute location of UAV *u* in global map.

(3) Auxiliary observation $O_{self}^{u}$: Then we utilize information from onboard flight control computer to assist UAV to learn optimal policy. Specifically, we define $O_{self}\;≜\;{\left\{o_{s}^{u}=\mathrm{concatenate}\left(x\left(u\right),y\left(u\right),v_{x}\left(u\right),v_{y}\left(u\right),\phi \left(u\right),{\left\{\Delta x(c),\Delta y(c)\right\}}_{\forall c\in C}\right)\right\}}_{\forall u\in U}\;$. For UAV *u*, $o_{s}^{u}$ includes its absolute position, velocity and current remaining battery, and the relative locations of all charging stations towards UAV *u*.

### 3.3. Action Space

The rotor UAVs are capable of applying different *thrust* at all directions responsively. We choose to discretize the entire 2-dimensional continuous space into eight directions for simplicity, and UAV agents can apply *maximum-thrust* (denoted as 1.0 unit), *half-thrust* (0.5 unit), or *zero-thrust* (0 unit) at any direction. Note that zero-thrust represents hovering in place. Therefore, the action space in SAG-MCS is defined as:

(2) $$A≜\left\{a_{t}^{u}=\left(\theta_{t}^{u},f_{t}^{u}\right)|\theta_{t}^{u}\in \left\{\frac{k\pi }{4}|k=0,1,\ldots ,7\right\},f_{t}^{u}\in \left\{0,0.5,1.0\right\}\right\}.$$

where $\theta_{t}^{u}$ denotes the thrust angle and $f_{t}^{u}$ is the thrust magnitude. The action space A consists of 17 actions in total. Since the timestep interval in the simulation is quite short, we assume the physical model is a uniform acceleration process. UAV can adjust the magnitude and direction of velocity using certain actions.

### 3.4. Evaluation Metrics

As stated in Section 3.1, the UAV swarm is aimed at collecting maximum information over PoIs as long as possible. UAVs should avoid collisions with obstacles and borders during movement, and recharge in time when power is low. Following Ye et al. [37] and Liu et al. [45], we propose three global evaluation metrics to evaluate the effectiveness of the joint cooperation of the UAV swarm in this SAG-MCS task. These metrics are ultimately used to evaluate the DRL policy we have trained.

The first metric is *Data Coverage Index*, which describes the average data amounts collected by the whole UAV swarm per timestep, as:

(3) $$c_{t}=\frac{\sum_{k=1}^{K}w_{t}\left(k\right)d\left(k\right)}{Kt},\;t=1,\ldots ,T.$$

where $w_{t}\left(k\right)$ denotes the number of timesteps when PoI *k* was successfully collected from timestep 1 till *t*. d(k) denotes the data amount carried by PoI *k* and *K* is the number of PoIs.

We noticed that in some cases, isolated PoIs in rural areas may not be covered even when the data coverage index is quite high; however, isolated or sparse PoIs in remote areas can carry valuable information in certain scenarios such as disaster relief. Considering the comprehensiveness of the data collection task, we propose the second global metric *Geographical Fairness Index* to evaluate the exploration ability of the UAV team, as:

(4) $$f_{t}=\frac{{\left(\sum_{k=1}^{K}w_{t}\left(k\right)d\left(k\right)\right)}^{2}}{K\sum_{k=1}^{K}{\left(w_{t}\left(k\right)d\left(k\right)\right)}^{2}},\;t=1,\ldots ,T.$$

where $w_{t}\left(k\right)$ and d(k) are defined the same as Equation (3). When all PoIs are evenly covered, Equation (4) gives $f_{t}=1$.

In addition, the third metric *Energy Consumption Index* is used to indicate the energy-saving status of the UAV swarm. In order to further simulate the energy consumed by multi-rotor UAV in reality, we adopt an equation of power on the flight speed [46], as:

(5) $$P_{T}=\frac{1}{2}C_{D}A\rho v^{3}+\frac{W^{2}}{\rho b^{2}v},$$

where $C_{D}$ is the aerodynamic drag coefficient, ρ is the density of air and *v* is the current flying speed. Parameter *A*, *W*, *b* denote UAV’s front facing area, total weight, and width, respectively. For simplicity, we adopt a general UAV model and specific values are omitted in this paper. In timestep *t*, we assume the consumed energy $e_{t}^{u}$ by UAV *u* is linear to its battery power, as:

(6) $$e_{t}^{u}=e_{0}+\eta_{e}{P_{T}}_{t}^{u},$$

where $e_{0}$ represents hovering energy consumption and $\eta_{e}$ is an energy coefficient. ${P_{T}}_{t}^{u}$ refers to the output power of UAV *u* in timestep *t*. Equations (5) and (6) reveal that UAV’s battery is more efficient at an optimal cruising flight speed, while hovering or flying at maximum speed will consume more power. Note that energy consumed during flight is mainly from rotors and embedded sensors, and we ignore the communication budgets in the ad-hoc network. Therefore, we define the energy consumption index by taking the average of all *U* UAVs in *T* timesteps:

(7) $$e_{t}=\frac{1}{t\times U}\sum_{\tau =1}^{t}\sum_{u=1}^{U}e_{\tau }^{u},\;t=1,\ldots ,T.$$

After a complete simulation episode, we calculate the metrics mentioned above as *final global metrics*, denoted as $\left\{c_{T},f_{T},e_{T}\right\}={\left\{c_{t},f_{t},e_{t}\right\}}_{t=T}$. We hope to maximize the coverage and fairness index for sensing data adequately, while minimize the energy consumption index for energy-saving. Therefore, following Ye et al. [37], we define the overall objective *coverage-fairness-energy score* (CFE score) by a DRL policy π:

(8) $${CFE}_{t}\left(\pi \right)=\frac{c_{t}\times f_{t}}{e_{t}},\;t=1,\ldots ,T.$$

Obviously, our objective is to optimize the policy π to maximize ${CFE}_{T}\left(\pi \right)$ of the whole episode. As our SAG-MCS is a practical partially observable scenario, UAV agents cannot be aware of these global metrics of the whole swarm. They can only make actions according to the decentralized policy $\pi_{u},\forall u\in U$ and self-owned information. Therefore, we propose a heuristic reward function to train the optimal policy π, which will be further introduced in Section 4.4.

## 4. Proposed ms-SDRGN Solution for SAG-MCS

**Figure 3:**
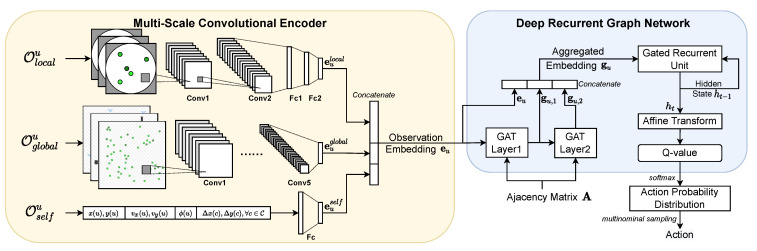
ms-SDRGN Model Architecture.

Due to the multi-scale observation space and complicated SAG-MCS task, we propose a heuristic DRL method named *Multi-Scale Soft Deep Recurrent Graph Network (ms-SDRGN)*. As illustrated in Figure 3, we first utilize a Multi-scale Convolutional Encoder to integrate local and global observed information for better feature extraction from observation space. Based on the concept of DRGN [37], we use graph attention mechanism (GAT [26]) to aggregate neighboring information through ad-hoc connections, and adopt gated recurrent unit (GRU [27]) as a memory unit for better long-term performance. In addition, we utilize a maximum-entropy method to learn stochastic policies via a configurable action entropy objective, and control each UAV agent in a distributed manner. Furthermore, a customized heuristic reward function is proposed for decentralized training.

### 4.1. Multi-Scale Convolutional Encoder

Exploiting observations properly is essential for agents to perceive the current state of RL systems and make corresponding actions. Previous DRL methods (e.g., DQN, DGN, MAAC) apply multi-layer perceptron (MLP) as linear encoders to process raw observations, which is preferred for scenarios with smaller observation dimensions or less information, such as Cooperative Navigation [47]. However, in our SAG-MCS task, observations and environment states are more complicated and their input sizes are relatively larger.

Our intuition lies that compared with MLP, convolutional neural network (CNN) is more capable of processing data that has spatial information and large receptive fields, such as images. CNN can integrate information from different input channels as well. So we treat the local observation $O_{local}$ and satellites’ fuzzy global observation $O_{global}$ as simplified real images, and design two CNN to extract spatial feature representations of local and global input states separately. Specifically, we construct the local CNN with two convolutional layers and two fully connected layers, which outputs local embedding $e_{u}^{local}$. The global CNN has a larger input scale, and we use five convolutional layers, which yields global embedding $e_{u}^{global}$. As for the auxiliary information in $O_{self}$, we simply use a fully connected layer and take $e_{u}^{self}$ as output from UAV self-owned information. Finally, we use concatenation operation to combine them as a multi-scale observation embedding $e_{u}$ for UAV *u*:

(9) $$e_{u}=\mathrm{concatenate}\left(e_{u}^{local}|e_{u}^{global}|e_{u}^{self}\right),\;\forall u\in U.$$

Such multi-scale features can help UAVs better select actions, by taking full account of: (a) the relative position between current UAV and surrounding PoIs, obstacles or other agents; (b) the correlation of current UAV’s remaining battery and the distance to the closest charging station; (c) the distribution of PoIs in the fuzzy global map for better exploration and coverage.

### 4.2. Aggregate Adjacent Information with Graph Attention Mechanism

For the purpose of multi-agents exchanging information through ad-hoc connections in SAG-MCS, we model the UAV swarm as a graph network, where each node is represented as a UAV, and the edges are the communication links of neighboring UAV pairs. For each node *i*, we denote $e_{i}$ extracted from observation space as its node embedding. Let all UAVs networked with UAV node *i* as a set $G_{i}$. This is implemented by an adjacency mask A, which is a U×U symmetric matrix and satisfies A(i,j)=1 if UAV node *i* is interconnected with UAV node *j*. For all UAV node $j\in G_{i}$, we utilize GAT to determine the weight of UAV node *i* towards its different neighbors *j* as $\alpha_{ij}$. Building on the concept of self-attention [48], an attention coefficient between node *i* and its neighboring node *j* is defined as $e_{ij}=a\left(We_{i},We_{j}\right)$, where a() is a shared attentional mechanism. Then, we calculate the attention weight $\alpha_{ij}$ by normalizing $e_{ij}$ across all possible node *j* using softmax function:

(10) $$\alpha_{ij}={\mathrm{softmax}}_{j}\left(e_{ij}\right)=\frac{\exp\left({\left(W_{K}e_{j}\right)}^{T}\cdot W_{Q}e_{i}\right)}{\sum_{k\in G_{i}}\exp\left({\left(W_{K}e_{k}\right)}^{T}\cdot W_{Q}e_{i}\right)},$$

Then GAT aggregates information from all adjacent nodes *j* by weighted summation, which is given by:

(11) $$g_{i}=\sum_{j\in G_{i}}\alpha_{ij}^{k}\cdot W_{V}e_{j}.$$

where we denote $g_{i}$ as the aggregated output embedding of UAV *j* after one GAT layer. In addition, $W_{Q},W_{K},W_{V}\in W$ are learnable weight matrics related with query, key, and value vector.

As shown in Figure 3, we utilize two GAT layers to aggregate information from neighboring UAV agents within a two-hop communication range, which could further expand the perception range and enhance cooperation of the UAV swarm. For better convergence, we then use skip connections [49] by concatenating the input observation embedding $e_{i}$, the outputs of the first GAT layer $g_{i,1}$ and the second GAT layer $g_{i,2}$, as $g_{i}=\mathrm{concatenate}\left(e_{i}|g_{i,1}|g_{i,2}\right)$.

Additionally, to make full use of temporal information during the interaction with RL environments and improve long-term performance, we integrate a gated recurrent unit (GRU) to memorize temporal features as:

(12) $$h_{t}=\mathrm{GRU}\left(g_{i}|h_{t-1}\right).$$

where we take $g_{i}$ as input and $h_{t}$ is the hidden state of timestep *t* stored in the memory unit. After adjacent information aggregation and GRU, we apply an affine transformation layer to $h_{t}$ for calculating Q-value $Q(O_{t},a_{t})$.

### 4.3. Learn Stochastic Policies with Adjustable Action Entropy

**Figure 4:**
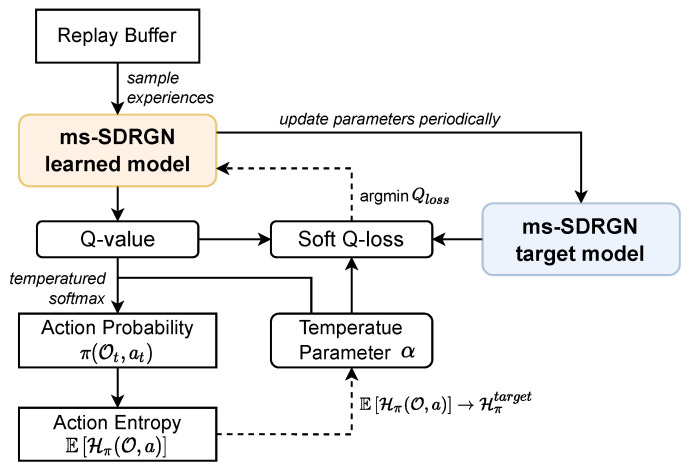
The training process of ms-SDRGN. In the flow chart, solid lines indicate feed forward propagation, and dashed lines denote updating parameters by backpropagation.

Based on the Q-value produced by DRGN, we can learn a deterministic policy, where each Q-value represents a fixed probability of the corresponding action. However, deterministic policies can easily jump into local optimum and lack for exploration in complex, real-world scenarios. Inspired by the maximum entropy RL framework [50,51], we utilize soft Q-loss to learn a stochastic policy in SAG-MCS, with the objective of maximizing expected reward and optimizing the action entropy towards a certain target. A flow chart of the training process is presented in Figure 4.

Firstly, we sample previous interaction experiences from the replay buffer as training inputs. The ms-SDRGN learned model infers a set of Q-value from the experiences. Then, we apply temperatured softmax operation to Q-value for getting the action probability:

(13) $$\pi \left(O_{t},a_{t}\right)={\mathrm{softmax}}_{a_{t}}\left(\frac{Q(O_{t},a_{t})}{\alpha }\right)=\exp\left(\frac{Q(O_{t},a_{t})}{\alpha }-\log\Sigma_{a_{t}}\exp\left(\frac{Q(O_{t},a_{t})}{\alpha }\right)\right),$$

where α is an adjustable temperature parameter, and Q-value $Q(O_{t},a_{t})$ is produced by the learned model when receiving $O_{t}$ and $a_{t}$ as inputs. Specific action during simulation is sampled from the action probability. Then, we use Equation (13) to estimate the action entropy by calculating the information entropy expectation from sampled experiences:

(14) $$E\left[H_{\pi }\left(O,a\right)\right]=E\left[-\Sigma_{a_{t}∼\pi }\pi \left(O_{t},a_{t}\right)\cdot \log\pi \left(O_{t},a_{t}\right)\right],$$

The action entropy represents the action uncertainty of policy π, which can be adjusted by the temperature parameter α. Therefore, we preset a target action entropy as $H_{\pi }^{target}=p_{\alpha }\cdot \max H_{\pi }$, where the maximum action entropy is determined by action space as $\max H_{\pi }=\log\left(\dim A\right)$, and $p_{\alpha }$ is a hyper-parameter named target entropy factor. Note that different RL tasks require different levels of exploration, so $p_{\alpha }$ shall be modified according to specific scenarios. More concretely, our goal is to let the action entropy $E\left[H_{\pi }\left(O,a\right)\right]$ approach the pre-defined target action entropy $H_{\pi }^{target}$, by updating the temperature parameter α through gradient descent:

(15) $$\nabla_{\alpha }=f\left(H_{\pi }^{target}-E\left[H_{\pi }\left(O,a\right)\right]\right).$$

where *f* is a customized activation function and $H_{\pi }^{target}$ denotes the target action entropy. The configurable action entropy mentioned above guarantees the balance between interaction stability and exploration capability of the policy.

Following Soft Q-learning [50], we also include the temperature parameter α to help define a V-value function for the target model. Finally, we use the mean squared error calculated by Q-value function and V-value function as $Q_{loss}$:

(16) $$V\left(O_{t}\right)=\alpha \cdot \log\Sigma_{a_{t}}\exp\left(\frac{Q(O_{t},a_{t})}{\alpha }\right),$$

(17) $$Q_{loss}=\frac{1}{S}\Sigma {\left(r_{t}+V\left(O_{t+1}\right)-Q\left(O_{t},a_{t}\right)\right)}^{2}.$$

where $r_{t}$ is the reward earned in timestep *t*, $V(O_{t})$ denotes the V-value function and *S* is the batch size. The Q-value function Q(o,a) of the learned model is updated through minimizing the $Q_{loss}$ in Equation (17). In the learning process, ms-SDRGN target model will be updated periodically by duplicating the parameters of the learned model directly.

### 4.4. Heuristic Reward Function

In this section, we design a heuristic reward function to evaluate the result when the UAV swarm conducted action $a_{t}$ based on respective observation $o_{t}$. Since each UAV agent in SAG-MCS is only exposed to local information and acts in a decentralized manner, we expect the reward function can help agents to achieve a better CFE score, while not directly aiming at optimizing the global metrics mentioned in Section 3.4. Therefore, the reward function considers the impact of data collection, battery charging, energy consumption and collision with boundaries.

Firstly, we encourage the UAV swarm to collect data as much as possible. Note that PoIs that within UAV’s coverage range $R_{cov}$ are referred as ‘covered’. For UAV *u*, we design an individual coverage term $r_{u}^{self}$ and a swarm coverage term $r_{u}^{swarm}$:

(18) $$r_{u}^{self}=\left\{\begin{array}{cc} \eta_{1}\cdot \Sigma_{p}d\left(p\right), & \mathrm{if}\;\mathrm{PoI}\;p\;\mathrm{is}\;\mathrm{covered}\;\mathrm{only}\;\mathrm{by}\;\mathrm{UAV}\;u\\ -1, & \mathrm{if}\;\mathrm{none}\;\mathrm{PoI}\;\mathrm{is}\;\mathrm{covered}\;\mathrm{by}\;\mathrm{UAV}\;u \end{array}\right.$$

(19) $$r_{u}^{swarm}=\left\{\begin{array}{cc} \frac{\eta_{2}}{n_{u}}\cdot \Sigma_{q}d\left(q\right), & \mathrm{if}\;\mathrm{PoI}\;q\;\mathrm{is}\;\mathrm{covered}\;\mathrm{by}\;\mathrm{other}\;\mathrm{UAVs}\;\mathrm{in}\;G_{u}\\ 0, & \mathrm{if}\;\mathrm{UAV}\;u\;\mathrm{is}\;\mathrm{not}\;\mathrm{networking}\;\mathrm{with}\;\mathrm{others} \end{array}\right.$$

where $r_{u}^{self}$ counts the data amounts collected individually by UAV *u*, and $r_{u}^{swarm}$ counts the data amounts covered by agents that network with UAV *u* in one-hop connection. They are expected to improve the data coverage index through both individual exploration and swarm cooperation. Let $n_{u}$ denote the number of UAV *u*’s one-hop neighboring nodes. In addition, we set balance coefficients $\eta_{1}=0.4$, $\eta_{2}=0.04$.

Secondly, in order to guide UAVs to charging stations when their batteries are low, we propose a charge term $r_{u}^{charge}$ as:

(20) $$r_{u}^{charge}=-\min\theta_{c}^{u},\;\forall c\in C,$$

where $\theta_{c}^{u}\in \left[0,1\right]$ is normalized euclidean distance between UAV *u* and charging station *c*. The charge term $r_{u}^{charge}$ will increase as UAV moving closer to its nearby charging station. We deem the UAV is in charging state when the relative distance meets $\theta_{c}^{u}\leq 2.0$, then an extra reward of 2.0 points will be added to $r_{u}^{charge}$.

Other factors such as energy consumption and collisions are considered as well. According to Equation (6), we simply define an energy term as $r_{u}^{energy}=1/e_{t}^{u}$. UAVs that consume less energy are expected to gain higher rewards. Then, we define a penalty term $p_{u}=1$ when UAV *u* collides with the fixed boundary in our scenario, otherwise put $p_{u}=0$.

We integrate local evaluation terms and define the heuristic reward function as:

(21) $$r_{u}=\frac{\left(r_{u}^{self}+r_{u}^{swarm}\right)\times \epsilon +r_{u}^{charge}\times \left(1-\epsilon \right)}{r_{u}^{energy}}+p_{u},\;\mathrm{if}\;\phi \left(u\right)>0,$$

where the weight parameter ϵ refers to the remaining battery percentage, denoted as ϵ=ϕ(u)/100%. Equation (21) only functions when battery is not empty, otherwise the reward function is defined as:

(22) $$r_{u}=r_{u}^{energy},\;\mathrm{if}\;\phi \left(u\right)\leq 0.$$

For training simplicity, UAV can still operate when its battery has drained, but it cannot get reward from data collection and will get an extra punishment.

## 5. Experiments

In this section, we introduce the setup of experiments and performance metrics. Then, we compare our approach with three state-of-the-art DRL baselines. Case studies are performed to analyze the effectiveness, expansibility and robustness of ms-SDRGN.

### 5.1. Experimental Settings

In this section, we use Pytorch 1.9.0 to perform experiments on Ubuntu 20.04 servers with two NVIDIA 3080 GPUs and an A100 GPU. In the SAG-MCS simulation environment, we set the 2D continuous target area of 200×200 pixels, where 120 PoIs, 3 charging stations, and 50 obstacles (20 round obstacles and 30 rectangular obstacles) are randomly initialized. PoIs are scattered around 3 major points from Gaussian distribution, each PoI is randomly assigned associated data within [1, 5]. We deploy 20 UAVs in the training stage with a parameter-shared model for action inference. We define their coverage range $R_{cov}=10$, the observation range $R_{cov}=13$, and the communication range $R_{comm}=18$ with the probability p=0.5 of communication dropout. The fuzzy global observation with the size of 40×40 pixels is updated from satellites to UAVs every 5 timesteps. Each UAV’s battery is initially fully charged to 100% and the consumed energy at each timestep is calculated after every movement, according to Equations (5) and (6).

In our implementation, the target entropy factor is set to $p_{\alpha }=0.3$ and the discounted factor γ is 0.99. We use Adam for optimization with the learning rate of 1 × 10${}^{-4}$, and ReLU as the activation function for all hidden layers. The experience replay buffer is initialized with the size of 2.5 × 10${}^{4}$ for storing interaction histories, and the batch size is set to 256. As for the exploration strategy, we apply ϵ−multinomial for stochastic policies such as ms-SDRGN, letting ϵ start with 0.9 and exponentially decay to 0 in the end. For deterministic policies, we use ϵ−greedy strategy and set ϵ to exponentially decay to 0.05 at 30,000 training episodes.

One simulation episode lasts for 100 timesteps, and each DRL model interacts with the simulation environment for 50,000 episodes in total. Interaction experiences will be pushed to the replay buffer concurrently. After each simulation episode, the learned network is trained for 4 times using the experiences sampled from the replay buffer, while the target network is updated every 5 episodes by directly copying the parameters from the learned network. After training, we test the converged models for 1000 episodes to reduce randomness.

As introduced in Section 3.4, we use the following metrics to evaluate the performance.

- *Episodic Reward*: calculates the accumulated reward of the whole evaluation episode. It generally evaluates the SAG-MCS task achievements by the UAV swarm, considering data collection, battery management and collisions.
- *Data Coverage Index* ($c_{T}$): describes the average data amount collected from PoIs.
- *Geographical Fairness Index* ($f_{T}$): shows how evenly the PoIs are covered by all UAVs geographically and represents the UAV swarm’s exploration level.
- *Energy Consumption Index* ($e_{T}$): calculates the average energy consumed by the UAV swarm, according to the flight speed and hovering status.
- *CFE Score* ($CFE_{T}$): represents the overall performance by combining $c_{T}$, $f_{T}$ and $e_{T}$ as Equation (8). We expect CFE score to be as large as possible.

### 5.2. Analysis of Training Convergence and Heuristic Reward Function

**Figure 5:**
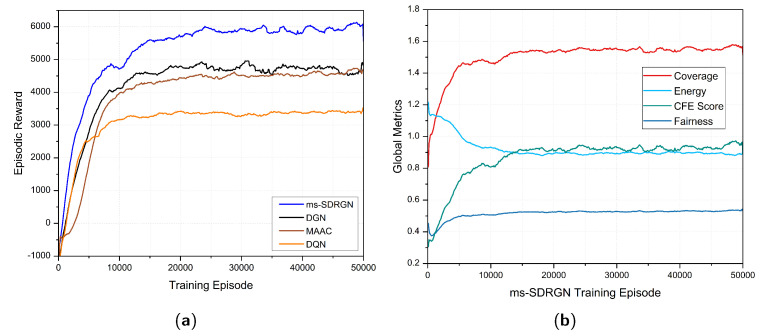
(**a**) The episodic reward learning curves of DRL algorithms. (**b**) The global metrics learning curves of ms-SDRGN.

To validate the feasibility and effectiveness of our SAG-MCS environment design and the heuristic reward function, we first present the learning curves of episodic reward and the global metrics over time. During the training phase, we evaluate the model for 20 episodes after every 100 training episodes, and calculate the average global metrics and accumulated reward, as illustrated in Figure 5.

In Figure 5a, we observe the average episodic reward of ms-SDRGN improves very quickly at the beginning, and gradually converges at around 20,000 episodes. Figure 5b presents the changes of four global metrics during the training progress of ms-SDRGN. The final energy index gradually drops and stabilizes to 0.9 at 20,000 episodes, indicating that UAVs have learned to operate at an optimal cruising speed. In addition, the final coverage and fairness index quickly grow and converge at around 10,000 episodes. Correspondingly, the overall CFE score has a similar growth trend and reaches convergence rapidly. Therefore, it can be proved that ms-SDRGN has learned the policy to fulfill the overall objective of maximizing the CFE score. After convergence, the UAV swarm can continuously collect PoIs maximumly using energy-efficient flying speed. The training results have suggested the effectiveness of the heuristic reward function.

Through visualization, we can observe that UAVs have learned to appropriately assign tasks at different remaining batteries. When its battery drops to around 25~40%, the UAV will proceed to the closest charging stations for battery exchange. In each simulation episode with 100 timesteps, the whole swarm rarely runs out of power, as such a charging process will happen two times for each UAV.

### 5.3. Comparing with DRL Baselines

We then compare our approach ms-SDRGN with three DRL baselines, including DGN [36], DQN [24] and MAAC [52]. DQN is a simple and efficient single-agent DRL approach, but it is still applicable for multi-agent tasks. Based on DQN, DGN uses GAT for modeling and exploiting the communication between agents. MAAC integrates self-attention mechanism with MADDPG [47], and provides agents with fully observable information to learn decentralized stochastic policy using a centralized critic. Thus, we compare ms-SDRGN with DGN to show the effectiveness of the multi-scale encoder and memory unit. Then, we compare with MAAC to validate the necessity of communication for the multi-agent swarm, especially in a partially observable environment.

We have evaluated the converged methods for 1000 episodes, and taken the mean value and standard deviation of all metrics, as shown in Table 1. Note that for a fair comparison, we also provide fuzzy global observations for the baselines, to ensure the raw observation inputs are the same.

The evaluation results are presented in Table 1. Then, we conduct a independent T-test between our approach and other three DRL baselines on every evaluation metric. It can be concluded that ms-SDRGN has a significant difference comparing to the baselines (p<0.05). We can obtain the following observations from Table 1:

Firstly, the proposed approach ms-SDRGN outperforms all other baselines in terms of reward and coverage index significantly. It demonstrates that with the help of multi-scale convolutional encoder and graph-based communication, ms-SDRGN achieves better data collection and energy management efficiency in SAG-MCS scenario. Compared with DQN and DGN, ms-SDRGN can better sense the surrounding environment from previous experiences in the memory unit, and make decisions more efficiently between seeking for more PoIs or returning for charging.

Secondly, from the perspective of fairness and energy, MAAC improves 0.005 fairness and 0.0075 energy index than ms-SDRGN. As a fully observable algorithm, we believe that MAAC can achieve similar cooperative exploration as ms-SDRGN using the observation embeddings from the whole UAV swarm. Regardless of extracting features from neighboring UAV nodes or from the memory unit, MAAC has a simpler objective to reduce its energy consumption for getting a higher reward.

Furthermore, the reward standard deviation of ms-SDRGN is higher than other methods, which may be attributed to randomness generated by the complex MADRL framework.

### 5.4. Analysis of Communication Dropout

**Figure 6:**
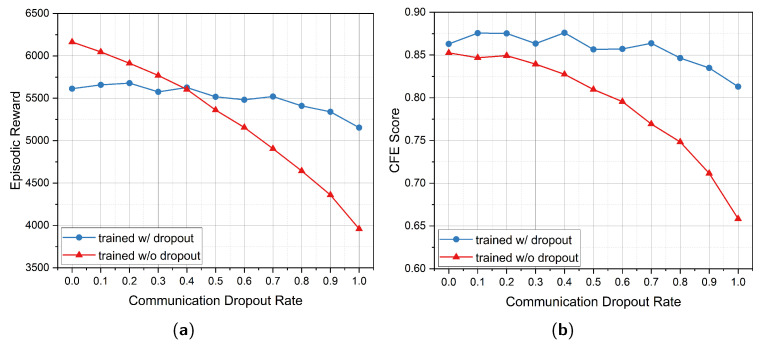
The evaluation results in environments with different communication dropout rate: (**a**) mean episodic reward and (**b**) CFE score.

In practical wireless networking applications, communication losses commonly occur in forms of delay, congestion or packet losses. To better cope with such real-world demanding communication conditions, we assume a p=0.5 probability of communication dropout between interconnected UAVs during the training phase. Theoretically, this setting can improve the robustness of our model when implemented in different conditions. Therefore, we have trained two ms-SDRGN models in environments with and without communication dropout, respectively. Then, we test them in SAG-MCS, where the random communication dropout rate *p* varies in [0,1], with an interval of 0.1. The evaluation result is shown in Figure 6.

From Figure 6a, it is observed that as the dropout rate grows in evaluation environment, the reward of the model trained w/o dropout continuously decreases. While the model trained w/ dropout achieves more stable evaluated reward and outperforms the other when the dropout rate *p* is larger than 0.4. In terms of the major metric CFE score in Figure 6b, ms-SDRGN trained w/ dropout continuously surpasses ms-SDRGN trained w/o dropout. When the evaluating communication dropout rate changes from 0 to 1.0, the CFE score of ms-SDRGN trained w/ dropout drops around 0.05 point. By contrast, ms-SDRGN trained w/ dropout gets 0.19 point of degradation on CFE score.

Random communication dropout can affect the stability of timing correlation in GRU memory unit. However, after trained in environment with 50% probability of communication losses, our ms-SDRGN is proved to be more robust, and will not result in significant performance loss even under unreliable communication conditions.

### 5.5. Impact of Simulation Environment Scale Setting

Next, we proceed to verify the performance of the multi-scale convolutional encoder. In actual MCS tasks, UAVs could encounter various densities of overground PoIs. For regions with dense PoI distributions such as modern cities, we hope to perform finer-grained observations for higher feature resolution. While we can perform coarse-grained or lightsized observations for areas with sparse PoIs. In order to handle tasks of different observation scales and enhance robustness, we implement CNNs as the multi-scale encoder, which technically is more applicable than linear encoders for large-scale observations. Therefore, we expect to compare the front-end multi-scale convolutional encoder with original linear encoder using different local observation scales.

In this experiment, we simulate different sizes of observation inputs by proportionally scaling the whole map, which could maintain the distribution of all elements and ensure comparison fairness. Specifically, we set the original environment setting introduced in Section 5.1 as scale 1.0 unit, and adjust the scale factor from 0.5 to 2.0 with the interval of 0.5 unit. The major settings of different scale factors are listed in Table 2.

**Figure 7:**
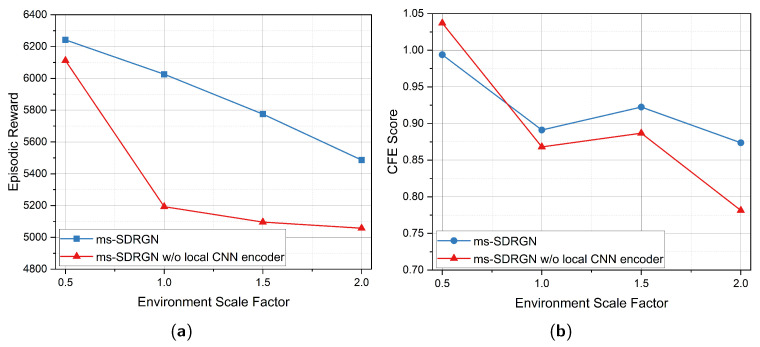
The evaluation results in environments with different scale factors: (**a**) mean episodic reward and (**b**) CFE score. (‘w/o local CNN encoder’ denotes using linear encoder to process local observations).

The evaluation results of four environment scales are presented in Figure 7. As size of local observation space varying with observation range $R_{obs}$, we can observe that CNN encoder outperforms linear encoder consistently on episodic reward. As for CFE score, ms-SDRGN with local CNN encoder achieves better CFE score than linear encoder when the scale factor is greater than or equal to 1.0, while linear encoder exceeds CNN encoder by 0.04 points at scale 0.5. The above result demonstrates that linear encoder can efficiently extract features from small-size input. In addition, the local CNN used in our multi-scale convolutional encoder has better representational capacity for large observation space. This finding demonstrates the expansibility of ms-SDRGN towards various scales of raw observations.

### 5.6. Ablation Study

Finally, we conduct an ablation study by separately removing components of ms-SDRGN, including multi-scale encoder, GAT layers, and GRU. We evaluate each case for 1000 episodes and the average results are listed in Table 3.

It can be observed in Table 3 that when removing any components, our ms-SDRGN will generally result in performance degradation. Firstly, removing local CNN encoder in case ‘-ms’ will reduce average CFE score and reward, which demonstrates the validity of CNN encoder, as discussed in Section 5.5. Secondly, case ‘-Soft’ demonstrates the stochastic policy outperforms the deterministic policy by improving exploration and coverage efficiency. Thirdly, case ‘-1GAT’ disables one GAT layer and limits the ad-hoc communication to one-hop range, which decreases 0.08 points on CFE score and 530 points on reward. Case ‘-2GAT’ disables both two GAT layers, which completely cuts off the communication of the UAV swarm and causes further performance loss. This finding suggests the necessity of GAT mechanism for modeling the communication between agents. Moreover, case ‘-GRU’ removes the memory unit and significantly reduces the average reward and CFE score. For complex MARL tasks such as SAG-MCS in this paper, the memory unit can help agents recall long-term experiences, especially when the positions of PoIs and obstacles are fixed.

## 6. Discussion

In this section, we discuss two limitations of our method and explore future directions for practical implementation.

Firstly, the computational complexity is crucial for practical applications. The proposed MADRL approach functions in a decentralized manner. Each UAV agent infers its action using on-board processor and executes the action subsequently. In addition, the multi-scale convolutional encoder introduced in Section 4.1 becomes the major computational burden for embedded processors. Therefore, future works will focus on introducing more computationally efficient spatial feature extractors.

Secondly, hand-crafted reward function limits the scalability. The heuristic reward function designed in Section 4.4 is customized for SAG-MCS simulation environment. When migrated to other application scenarios, the reward function requires modification case to case. Inverse reinforcement learning can be a solution for agents to infer reward functions from expert trajectories [53].

## 7. Conclusions

This paper introduced a partially observable MCS scenario named SAG-MCS, with an aerial UAV swarm jointly performing data collection task under energy limits. We proposed a value-based MADRL model named ms-SDRGN to address this multi-agent problem. Conclusively, ms-SDRGN applied a multi-scale convolutional encoder to handle the multi-scale observations, and utilized GAT and GRU for modeling communications and providing long-term memories. Effectively, a maximum-entropy method with configurable action entropy was employed to learn a stochastic policy. Experiments were conducted to demonstrate the superiority of our model compared with other DRL baselines, and validate the necessity of major components in ms-SDRGN. In addition, we analyzed the effectiveness of the communication dropout setting and the front-end CNN encoder. Future works will be focused on implementing fully continuous action space and exploring multi-stage multi-agent scenarios.

## Figures and Tables

**Table 1:** Comparison of DRL Baselines.

Algorithm	Reward	CFE Score	Coverage	Fairness	Energy
ms-SDRGN	6025.82±911.13	0.8911±0.1699	1.5345±0.1509	0.5205±0.0423	0.9067±0.0347
DGN	4730.38±840.32	0.8104±0.1802	1.4636±0.1600	0.5032±0.0514	0.9226±0.0514
MAAC	4670.71±783.61	0.8587±0.1799	1.4496±0.1519	0.5255±0.0484	0.8992±0.0373
DQN	3291.03±631.22	0.6273±0.1367	1.3332±0.1554	0.5027±0.0521	1.0819±0.0227

**Table 2:** Simulation Environment Scale Experiment Settings.

Environment Scale Factor	0.5	1.0	1.5	2.0
Environment Size in Pixels	100×100	200×200	300×300	400×400
Coverage Range $R_{cov}$	5	10	15	20
Observation Range $R_{obs}$	7	13	20	26
Communication Range $R_{comm}$	9	18	27	36

**Table 3:** Ablation study of ms-SDRGN method.

Algorithm	Reward	CFE Score
ms-SDRGN	6025.82±911.13	0.8911±0.1699
ms-SDRGN-ms	5198.48±877.04	0.8674±0.1985
ms-SDRGN-Soft	5337.79±814.41	0.8592±0.1791
ms-SDRGN-1GAT	5523.05±905.01	0.8102±0.1762
ms-SDRGN-2GAT	4984.57±837.96	0.7956±0.1729
ms-SDRGN-GRU	4318.14±815.62	0.8017±0.1878

‘-ms’ means removing local CNN encoder. ‘-Soft’ means training a deterministic policy instead of a stochastic policy. ‘-1GAT’ and ‘-2GAT’ denotes disabling one GAT layer and two GAT layers separately. ‘-GRU’ means disabling GRU memory unit.

## Data Availability

Not applicable.

## Abbreviations

The following abbreviations are used in this manuscript:

MCS	Mobile Crowdsensing
SAG-MCS	Space-Air-Ground integrated Mobile CrowdSensing
DRL	Deep Reinforcement Learning
MADRL	Multi-Agent Deep Reinforcement Learning
UAV	Unmanned Aerial Vehicle
PoI	Point of Interest
POMDP	Partially Observable Markov Decision Process
ms-SDRGN	Multi-Scale Soft Deep Recurrent Graph Network
GAT	Graph Attention Network
GRU	Gated Recurrent Unit
CFE	Coverage-Fairness-Energy Score
CNN	Convolutional Neural Network
MLP	Multi-Layer Perceptron
